# Carcinome neuroendocrine à grandes cellules primitif du sein: une tumeur rare chez l’homme

**DOI:** 10.11604/pamj.2016.25.205.10366

**Published:** 2016-11-29

**Authors:** Fatima Safini, Zineb Bouchbika, Zineb Bennani, Sara Belkheiri, Hicham El Attar, Nadia Benchakroun, Hassan Jouhadi, Nezha Tawfiq, Souha Sahraoui, Abdellatif Benider

**Affiliations:** 1 Service d’Oncologie et de Radiothérapie, CHU Ibn Rochd, Casablanca, Maroc; 2 Laboratoire d’Etude Anatomopathologique Moulay Driss Premier, Casablanca, Maroc

**Keywords:** Cancer du sein, neuroendocrine, grandes cellules, Breast cancer, neuroendocrine, large cells

## Abstract

Le carcinome neuroendocrine à grandes cellules primitif du sein est une entité extrêmement rare. Moins de dix cas ont été rapportés dans la littérature. Nous présentons un nouveau cas survenu chez un homme de 61 ans qui s’est présenté pour une tumeur localement avancée du sein droit d’emblée métastatique aux poumons et à la plèvre, classée cT4bN1M1. Le patient avait reçu huit cycles de chimiothérapie à base de Docetaxel tous les 21 jours, avec une bonne réponse clinique et radiologique (>50%), puis mis sous hormonothérapie à base de tamoxifène avec une stabilisation pendant 18 mois. L’étude immuni-histochimique reste indispensable pour déterminer la nature neuroendocrine de cette tumeur. Le traitement n’est pas bien codifié vu la rareté de ce type de cancer.

## Abstract (en)

Primitive large cell neuroendocrine tumor of the breast is an extremely rare entity. Less than ten cases have been reported in the literature. We report a new case of a 61-year old male patient presenting with locally advanced tumor of the right breast, immediately metastasized to the lungs and pleura, staged cT4bN1M1. The patient received eight cycles of Docetaxel-based chemotherapy every 21 days, with a good clinical and radiological response (>50%), followed by adjuvant tamoxifen with disease stabilization for 18 months. Immunohistochemistry is still essential to determine the neuroendocrine nature of this tumor. Treatment is not well codified due to the rarity of this type of cancer.

## Introduction

Le cancer du sein chez l’homme représente moins de 2% de l’ensemble des cancers masculins. Le type anatomopathologique le plus fréquent est le carcinome canalaire infiltrant (90%). Les tumeurs neuroendocrines (TNE) mammaires sont exceptionnelles et seulement une dizaine de TNE du sein chez l’homme sont rapportées [1]. Le sous-groupe à grandes cellules est une entité plus rare avec moins de dix cas rapportés chez la femme alors qu’il n’a pas été décrit de carcinome neuroendocrine (CNE) à grandes cellules primitif du sein chez l’homme. Nous rapportons le premier cas.

## Patient et observation

**Figure 1:**
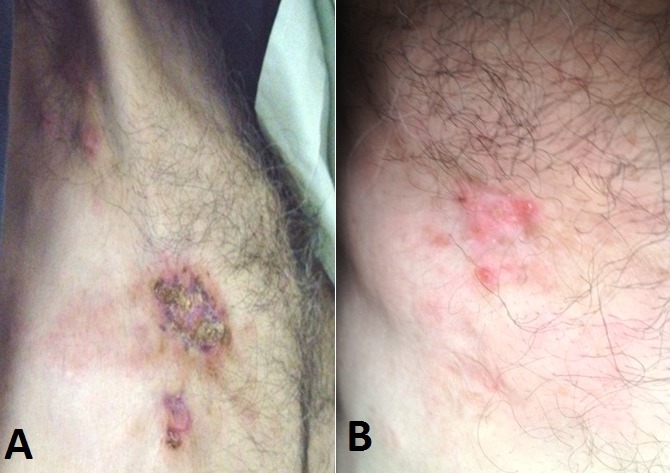
A) le mamelon droit complètement détruit et ulcéré, avec plusieurs nodules de perméation; B) réponse clinique après chimiothérapie avec disparition des lésions

**Figure 2:**
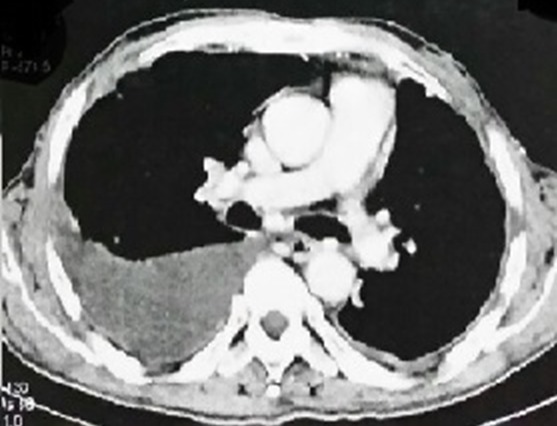
Coupe scannographique thoracique montrant l’épanchement pleural en fenêtre médiastinale

**Figure 3:**
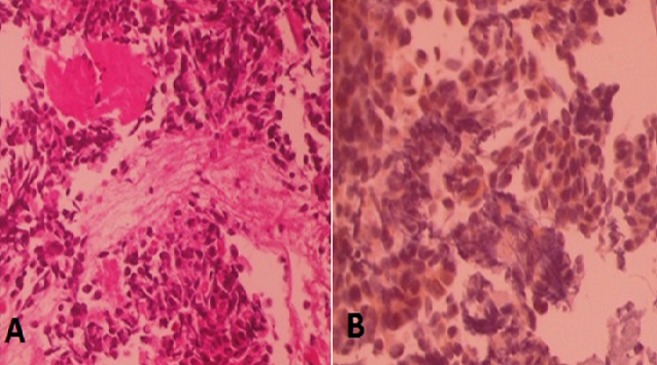
A) coloration à l’hémateine eosine au grossissement 200: prolifération tumorale maligne à cellules rondes de grande taille; B) étude immuno-histochimique au grossissement 400: expression de la chromogranine par les cellules tumorales

Mr K.M, âgé de 61 ans, marié et père de 4 enfants, sans antécédents pathologiques particuliers, s’est présenté pour une toux sèche associée à une dyspnée intense évoluant dans un contexte d’altération de l’état général et d’amaigrissement important chiffré à 15 Kg en 2mois. L’anamnèse a détecté dans son histoire de la maladie l’apparition un an auparavant d’un nodule du sein droit augmentant progressivement de taille, négligé, ayant évolué vers l’ulcération de la peau en regard. L'examen clinique, avait retrouvé un syndrome d’épanchement thoracique droit. Par ailleurs, le sein droit était complètement détruit et ulcéré, entouré par plusieurs nodules de perméation, associé à des adénopathies axillaires palpables homolatérales mobiles (Figure 1 A). Une radiographie thoracique réalisée dans ce contexte avait objectivé un épanchement pleural de moyenne abondance à droite. Nous avons complété par un scanner thoraco-abdominal qui avait également retrouvé un épanchement pleural à droite avec multiples lésions nodulaires et micronodulaires disséminées aux deux champs pulmonaires (Figure 2) sans objectiver de lésions suspectes au niveau du foie. A la ponction biopsie pleurale, il s’agissait d’une prolifération tumorale maligne indifférenciée à grandes cellules (Figure 3 A). L’étude immuno-histochimique avait objectivé une positivité des cellules tumorales aux anticorps anti-cytokératine, anti-chromogranine et anti-synaptophysine (Figure 3 B). Une biopsie du sein droit a été également réalisée et qui avait conclu à un carcinome neuro-endcrine à grandes cellules primitif du sein devant la positivité en plus des marqueurs neuroendocrines, des récepteurs hormonaux (récepteurs oestrogéniques étaient exprimés à 90% et les récepteurs progestatifs à 90%). Le marquage de l’HER2 était négatif. Le bilan d’extension a été complété par une scintigraphie osseuse n’ayant pas objectivé d’autres localisations secondaires. Le diagnostic retenu était celui d’un carcinome neuroendocrine à grandes cellules primitif du sein classé cliniquement cT4bN1M1. Le patient avait reçu huit cycles de chimiothérapie selon le protocole Docetaxel 75mg/m2 tous les 21 jours. La chimiothérapie a été bien tolérée. La réponse tumorale clinique a été estimée à plus de 90% au niveau local (Figure 1 B) et à 75 % au niveau des métastases pleuro-pulmonaires. Une hormonothérapie de maintenance anti-estrogénique de type tamoxifène a été prescrite. Avec un recul de 18 mois de la fin du traitement, le patient est maintenu en situation de contrôle de la maladie locale et métastatique. Un traitement local a été discuté à plusieurs reprises (chirurgie et/ou radiothérapie) mais qui était refusé par le patient.

## Discussion

Plus de 97% des tumeurs neuroendocrines surviennent au niveau pulmonaire et du tractus gastro-intestinal, alors que les tumeurs neuroendocrines primitives du sein sont rares et représentent 2 à 5 % des cancers mammaires [2]. Peu de cas ont été rapportés au niveau du sein chez la femme, et aucun cas n’a été rapporté chez l’homme. Ces tumeurs sont définies par leur morphologie similaire aux tumeurs neuroendocrines développées au niveau des autres sites et par l’immuno-expression de marqueurs neuroendocrines dans plus de 50 % du volume tumoral. En 2003, l’Organisation Mondiale de la Santé (OMS) a distingué quatre sous-types de CNE du sein: les carcinomes neuroendocrines solides, les carcinoïdes atypiques, les carcinomes à petites cellules et les carcinomes neuroendocrines à grandes [3]. Les carcinomes neuroendocrines à grandes cellules sont des tumeurs peu différenciées de haut grade de malignité [4, 5]. À l’examen histologique, le diagnostic de la nature neuroendocrine de ces tumeurs est évoqué à la morphologie et confirmé après une étude immunohistochimique à l’aide de marqueurs neuroendocrines, essentiellement la chromogranine A, la synaptophysine, la neuron-specific enolase (NSE) et le CD56 [4, 6]. Cependant, l’expression des marqueurs neuroendocrines est inconstante et l’absence d’expression de l’un de ces marqueurs n’exclu pas le diagnostic de CNE du sein. Dans les sept cas mammaires publiés dans la littérature, la synaptophysine était toujours exprimée alors que la chromogranine n’était positive que chez 4 cas (Tableau 1). Dans notre observation, la synaptophysine et la chromogranine étaient exprimées, aussi bien que les récepteurs d’estrogène et de progesterone. Les récepteurs hormonaux sont rarement présents dans les CNE du sein et peuvent rendre le pronostic plus favorable [5]. Ce qui explique la bonne évolution chez notre patient. Néanmoins, leur expression dans le sein n’est pas pathognomonique de l’origine mammaire. Le diagnostic d’un CNE à grandes cellules primitif du sein ne doit être retenu qu’après avoir éliminé un autre foyer primitif ou en cas de présence d’une composante canalaire in situ associée [4]. L’expression des CK7 et CK20 peut aussi être utile pour l’orientation diagnostique. La CK7 est largement exprimée dans les tumeurs épithéliales d’origine pulmonaire, salivaire, endométriale et mammaire. La CK20 est surtout exprimé dans les cancers du côlon, du pancréas et les tumeurs à cellules de Merkel cutanées. L’expression coordonnée de ces deux marqueurs peut aussi être utilisée dans le CNE du sein pour éliminer un site primitif autre que mammaire [7]. Vu la rareté de cette entité, il n’existe pas de traitement bien codifié. La plupart des données publiées ont été extraites des séries publiées sur les CNE du sein tous types confondus. Les CNE du sein sont traités par les uns comme un adénocarcinome du sein et par d’autres comme un carcinome neuroendocrine du poumon [5]. Les carcinomes neuroendocrines à variante solide et les carcinoïdes atypiques semblent avoir un meilleur pronostic par rapport aux carcinomes neuroendocrines à petites cellules et les carcinomes peu différenciés à grandes cellules [4]. Bien que le pronostic des CNE à grandes cellules du poumon reste défavorable avec un risque élevé de métastases à distance, au niveau du sein, son pronostic reste difficile à déterminer du fait du caractère rare de cette tumeur [5]. Mais d’après les données de la littérature, les CNE à grandes cellules du sein semblent avoir un pronostic favorable [8].

**Tableau 1:** Les cas de CNE à grandes cellules publiés dans la littérature

Auteurs	Age (ans)	Sexe	RE	RP	HER2	Chromo	Synapto	NSE	Classification (TNM)	Traitement	Evolution
Kim et al (2008)	27	femme	ND	ND	ND	(+)	(+)	(+)	ND	CHT adjuvante (type non spécifié)	RC à 18 mois
Boughaleb et al (2009)	28	Femme	(-)	(+)	ND	(-)	(+)	ND	T4dN1M0	CHT néoadjuvante (4AC60). Chirurgie CHT adjuvante (6 Etoposide/cisplatine) RTH paroi+sus-clav+CMI. HT (tamoxifène)	RC à 12 mois
Okoshi et al (2012)	63	Femme	(-)	(-)	(-)	(-)	(+)	(+)	pT2N0M0	Chirurgie RTH adjuvante Uracil+tegafur	RC à 44 mois
Psoma et al (2012)	46	Femme	ND	ND	ND	(+)	(+)	ND		Chirurgie (mastectomie+curage ganglionnaire) CHT adjuvante (Etoposide/cisplatine/epirubicicne)	RC à 6 mois
Yoshimura et al (2015)	34	femme	(-)	(-)	(-)	(+)	(+)	(+)	ND	Chirurgie : Patey Pa s traitement adjuvant	RC à 48 mois
Janosky et al (2015)	34	Femme	(-)	(-)	(-)	(+)	(+)	ND	cT2N0M0	CHT néoadjuvante (4 AC+ 4paclitaxel). Chirurgie	Progression locale et métastatique (os+poumon) juste après chirurgie
Notre cas	61 ans	Homme	(+)	(+)	(-)	(+)	(+)	ND	T4bN1M1	CHT (6 cycles docetaxel). HT (tamoxifène)	Contrôle de la maladie locale et métastatique à 18 mois.

RE: récepteurs oestrogéniques ; RP: récepteurs à la progesterone; chromo: chromogranine, synapto: synaptophysine; (+): positif; (-): négatif; CHT: chimiothérapie; RTH: radiothérapie, HT: hormonothérapie, ND: non disponible

## Conclusion

Le carcinome neuroendocrine à grandes cellules primitif du sein est une tumeur extrêmement rare chez l’homme. Vu sa rareté, il n’existe pas de standard thérapeutique et le pronostic demeure difficile à déterminer. La reconnaissance de cette entité est indispensable pour adapter une prise en charge optimale.
